# The First Report of an Intraperitoneal Free-Floating Mass (an Autoamputated Ovary) Causing an Acute Abdomen in a Child

**DOI:** 10.1155/2012/615734

**Published:** 2012-10-15

**Authors:** Ibrahim Uygun, Bahattin Aydogdu, Mehmet Hanifi Okur, Selcuk Otcu

**Affiliations:** Department of Pediatric Surgery and Pediatric Urology, Medical Faculty of Dicle University, 21280 Diyarbakir, Turkey

## Abstract

A free-floating intraperitoneal mass is extremely rare, and almost all originate from an ovary. Here, we present the first case with an intraperitoneal free-floating autoamputated ovary that caused an acute abdomen in a child and also review the literature. A 4-year-old girl was admitted with signs and symptoms of acute abdomen. At surgery, the patient had no right ovary and the right tube ended in a thin band that pressed on the terminal ileum causing partial small intestine obstruction and acute abdomen. A calcified mass was found floating in the abdomen and was removed. The pathological examination showed necrotic tissue debris with calcifications. An autoamputated ovary is thought to result from ovarian torsion and is usually detected incidentally. However, it can cause an acute abdomen.

## 1. Introduction

An autoamputated ovary (AO) is a very rare cause of an intraabdominal mass [1–25]. The primary pathological event of an AO is torsion of a normal ovary or an ovarian cyst and the adnexa, followed by infarction and necrosis [17, 21, 26, 27]. Typically, the AO is found incidentally while investigating an unrelated disease, on antenatal ultrasonography, or at surgery [1–25].

Here, we present a patient who underwent surgery for an acute abdomen and was observed to have a free-floating AO in the abdominal cavity. We also review the occurrence of this extremely rare free-floating mass in children and discuss its diagnosis and management.

## 2. Case Presentation

**Figure 1:**
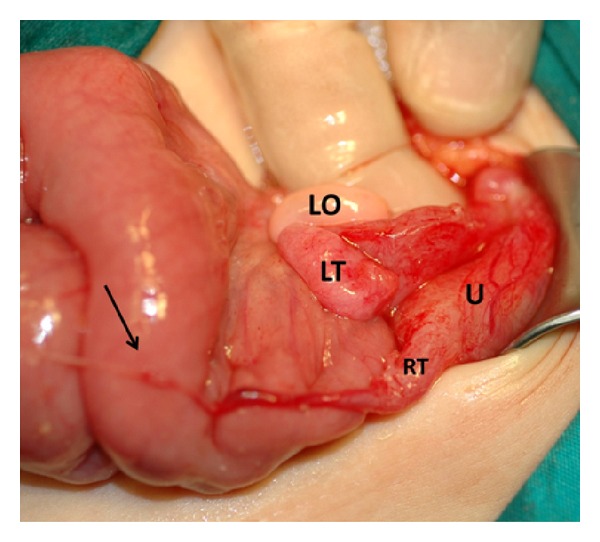
The right tube (RT) ended in a thin band (arrow) attached to the cecum and pressed on the terminal ileum. The patient had no right ovary. The left ovary (LO) and tube (LT) and uterus (U) were normal.

**Figure 2:**
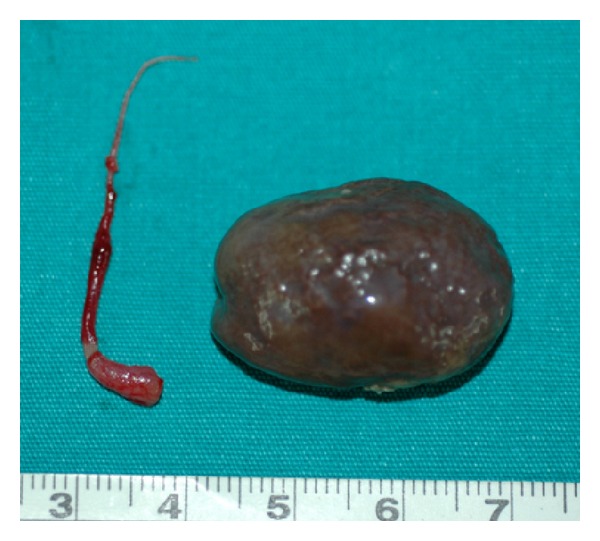
The brown, soft, calcified free-floating autoamputated right ovary (right) and the excised right tube ending with a thin band (left).

A 4-year-old girl was admitted with nausea, vomiting, and abdominal pain. On physical examination, the right lower abdominal quadrant was tender. Abdominal guarding and rebound were detected. The abdominal plain X-ray was normal. Emergency ultrasonography (US) showed minimal free fluid. The patient underwent surgery for an acute abdomen. At surgery, a 28 mm diameter, brown, soft, calcified mass was found floating in the right lower abdomen (Figure 1). The patient had no right ovary and the right tube ended in a thin band that extended to the cecum and pressed on the terminal ileum causing partial small intestine obstruction and acute abdomen (Figure 2). The appendix was hyperemic. The free-floating mass was removed from the abdomen, the right fallopian tube and band were excised, and an appendectomy was performed. The patient was discharged on the second postoperative day. The pathological examination showed long standing necrotic tissue debris with calcifications. The 6-year follow up showed no problems.

## 3. Discussion

A free-floating intraperitoneal mass is extremely rare, and almost all originate from an ovary. To date, there have been only two cases in the literature that originated from other organs [28, 29]; one such mass in a geriatric woman was from the gallbladder, due to torsion, and caused acute abdomen, while the other was from appendix epiploica, due to torsion, in a man [28, 29]. A free-floating intraperitoneal AO in a child was first reported by Lester and McAlister in 1970 [1]. Our case is the first report of an intraperitoneal free-floating mass causing an acute abdomen in a child.

There have been only 36 reported cases of intraperitoneal free-floating AO involving children ranging in age from 1 day to 12 years of age, including our case (Table 1) [1–25]. Twenty-five cases were younger than 1 year of age. Although 23 of these infants were diagnosed with a cystic abdominal mass, ranging in diameter from 2.2 to 8 cm on antenatal US, only 12 of the newborns were operated on during the neonatal period.

Six cases were symptomatic, including our case. One of the newborns had abdominal distention, intestinal obstruction, and respiratory distress syndrome due to an 8 cm diameter cyst [24]. Four children, ages 14 and 17 months, 2 years, and 12 years, had a history of abdominal pain without an acute abdomen and were diagnosed during routine physical examinations [1, 3, 7]. Only our 4-year-old patient developed an acute abdomen, with signs and symptoms that included tenderness in the right abdominal quadrant, nausea, and vomiting.

Nine of the masses could be palpated on physical examination. Only three cases were diagnosed as an AO preoperatively. Characteristically, an AO is seen as a free-floating intraabdominal mass on antenatal US [13, 19, 25]. Eight cases were diagnosed incidentally. Two had no abdominal pain but were diagnosed based on palpating an abdominal mass during a routine physical examination [3]. The other six patients were diagnosed with a calcified mass seen on plain X-rays obtained for an unrelated reason [2, 3, 6, 10, 12]. The AO was the right ovary in 17 cases, the left in 11, bilateral in two, and unknown in six cases.

Ultrasonography is safe and sufficient for diagnosing most ovarian cysts and AO. Computed tomography and magnetic resonance imaging may be performed if the mass is complex [22]. In our case, emergency US showed minimal free fluid, but no AO, perhaps because our patient had an intestinal obstruction and a dilated intestine with intraluminal gas. A plain X-ray may also be sufficient, especially with a calcified AO [1–3, 5, 7, 10]. In the literature, 25 of 36 cases of AO were diagnosed prenatally with antenatal US. We believe that this is because antenatal US is performed very commonly worldwide.

Pathologically, necrosis was seen in all cases, and 20 had calcifications. Small amounts of ovarian tissue were seen in seven specimens [3, 7, 18, 20, 23, 25]. In four of these cases, the AO was attached to the retroperitoneum and ascending colon by vessels [3], the omentum [23], the mesentery of the transverse colon via a long pedicle [7], or to the liver via a hemorrhagic twisted pedicle of omentum [7]. None contained malignant tissue. In adults, Ushakov et al. reported a remarkable characteristic of AO: an AO teratoma became reimplanted as an omental mass in 22 cases of teratoma of the omentum that they reviewed [26]. This adult review and our review of children suggest that an AO may reimplant, develop into omentum or peritoneum, and possibly undergo malignant transformation. Therefore, we suggest that all AOs should be excised instead of taking a wait and see approach.

An AO is very rare and thought to result from ovarian torsion. Most free-floating AOs are detected incidentally. However, clinicians should remember that it can cause an acute abdomen, and should always make sure there are two ovaries on US in a small child with acute abdomen.

## Figures and Tables

**Table 1:** Cases of autoamputated free-floating ovaries in children.

No.	Reference	Age	Clinical and imaging features	Size (cm)	Side	Surgical finding	Treatment	Pathology
1	[1]	12 y	Abdominal pain, no PM, PXR, mobile pelvic calcified mass	3.0 × 2.2	R	Absent RO and partial RFT, calcified FFM	LT	Solid NT with calcification
2	[2]	3 y	Incidental calcification on IVU for UTI, asymptomatic, no PM, PXR, mobile pelvic calcified mass	UK	R	UK	UK	UK
3	[2]	4 y	Incidental calcification on hip X-ray for lower extremity pain, asymptomatic, no PM, PXR, mobile pelvic calcified mass	UK	L	UK	UK	UK
4	[3]	17 m	Mobile fluctuant nontender mass on a routine PE, history of colic pain, PM, PXR, calcified mass, IVU, normal	5.0	R	Absent RO and RFT, cystic greenish-brown FFM attached to the omentum	LT	NT, fibrosis, hemorrhage, calcification, no VOT
5	[3]	5 m	Soft nontender mass on a routine PE, asymptomatic, PM, PXR, irregular calcification in the lower abdomen, IVU, normal	4.0 × 3.0	R	Absent RO, rudimentary RFT, cystic FFM attached to the omentum	LT	Calcified fibrous NT
6	[3]	9 y	Incidental calcification in PXR for undetermined reason, asymptomatic, no PM, PXR, calcification, IVU, normal	3.0 × 2.3	R	Absent RO, calcified FFM, normal fallopian tubes and LO	LT	NT with calcification, no VOT
7	[3]	2 w	Mobile nontender mass on a routine PE, asymptomatic, PM, IVU and barium enema, normal	3.0 × 1.5	R	Absent RO and RFT, cystic FFM attached to the retroperitoneum and ascending colon	LT	Ovarian stroma and follicles, calcified NT, fibrous wall
8	[4]	3 m	CM in AUS at 38-GW, asymptomatic, no PM, US, mobile CM	4.0	R	Absent RO, atretic RFT, cystic FFM	LT	NT, fibrotic walls, no VOT
9	[5]	4 d	CM in AUS at 38-GW, asymptomatic, no PM, lower abdominal fullness, PXR, noncalcified mass, US, CM	8.0	R	Absent RO, hemorrhagic cystic FFM	LT	NT
10	[6]	2 y	Incidental bilateral pelvic calcification in IVU for recurrent UTI, asymptomatic, no PM, US and CT, bilateral pelvic calcifications	3.5 × 2.5 2.5 × 2.0	BL	Two FFMs in the cul-de-sac, absent ovaries, normal uterus, and fallopian tubes	LT	Extensive NT and calcification
11	[7]	2 y	Recurrent abdominal pain, PM, abdominal tenderness, PXR, soft tissue mass with calcification, US, CM with solid component	6.5	R	Absent RO and RFT, cystic FFM attached through a long pedicle to mesentery of colon	LT	Unilocular cyst filled with thick fluid with calcified mural nodule
12	[7]	14 m	Right lower quadrant mass on PE for abdominal pain, PM, PXR, calcified mass, US, CM with a solid mural nodule	5.0 × 4.0	R	Absent RO and RFT, cystic FFM attached to liver through twisted pedicle of omentum	LT	CM, shaggy tan-pink interior with gritty mural nodule
13	[8]	2 w	CM in AUS at 26-GW, asymptomatic, no PM, US, CM in the right upper quadrant, fluid-fluid level	6.0 × 6.0	R	Absent RO, cystic FFM	LT	Aseptic necrosis of ovary with pseudocyst formation
14	[9]	5 m	CM in AUS at 30-GW, asymptomatic, no PM, US, complex ovarian cyst with calcification	6.5	UK	Autoamputated cystic ovarian FFM	LT	UK
15	[9]	7 m	CM in AUS at 34-GW, asymptomatic, no PM, US, complex ovarian cyst with fluid debris level	3.0	UK	Autoamputated cystic ovarian FFM	LT	UK
16	[10]	8 y	Incidental calcification on a PXR obtained for UTI, asymptomatic, no PM, PXR, CT and VCUG, calcification adjacent to the pubic bone	3.0 × 3.0	R	Absent RO, calcified FFM	LT	Necrotic partially calcified mass, no VOT
17	[11]	11 m	CM in AUS at 34-GW, asymptomatic, freely mobile PM, US, ovarian cyst with fluid/debris level	4.0	R	Absent RO and RFT, cystic FFM	LT	NT, no VOT
18	[12]	6 y	Incidental calcified mass on a PXR obtained for coin ingestion, asymptomatic, no PM, US, absent LO, CT, mobile calcified pelvic mass	2.2 × 1.7	L	Absent LO and LFT, calcified FFM	LS	Amorphous calcified tissue, no VOT
19	[13]	5 m*	CM in AUS at 38-GW, asymptomatic, mobile PM, US, right-sided cystic pelvic mass with echogenic finding	4.2 × 3.7	L	Atretic LFT covered with peritoneum, cystic FFM	LS	Extensive NT and autolysis with calcification
20	[14]	Neonate	UK	UK	UL	Autoamputated cystic ovarian FFM	LS	UK
21	[15]	6 w	CM in AUS, asymptomatic, no PM, US, hemorrhagic RO cyst	4.0 × 3.0	R	Absent RO and RFT, cystic FFM attached to the omentum	LS	Hemorrhagic infarction with calcification
22	[16]	5 m	Two CM in AUS at 17-GW, asymptomatic, no PM, US, two CM with septations	3.5 × 2.5 3.8 × 1.8	BL	No ovaries, two cystic FFMs, normal uterus and fallopian tubes	LT	Hemorrhagic ovaries with calcification
23	[17]	Infant	Asymptomatic, no PM, US, CM	UK	UL	Cystic FFM	LS	No VOT
24	[18]	3 m	CM in AUS at 27-GW, asymptomatic, no PM, US, ovarian cyst with fluid debris level	5.0	UK	Cystic FFM	LT	Ovarian NT, hemorrhage, calcification
25	[19]	4 w*	CM in AUS at 32-GW, asymptomatic, no PM, US, CM in the right side	4.5	L	Absent LO and LFT, hemorrhagic cystic FFM adhered loosely to peritoneum	LS	No VOT, hemorrhagic NT
26	[20]	3 m	CM in AUS at 24-GW, asymptomatic, no PM, US, pelvic CM	4.0 × 3.5	UK	Autoamputated cystic ovarian FFM	LT	Cystic ovary with NT
27	[21]	3 w	CM in AUS at 30-GW, asymptomatic, no PM, US and CT, CM with calcification, MR, hemorrhagic mass	3.2 × 2.0	R	Absent RO and RFT, cystic FFM	LT	NT, hemorrhage, autolysis with calcification, no VOT
28	[22]	2 d	CM in AUS at 32-GW, asymptomatic, mobile PM, US, a free-floating CM without blood support with fluid/debris level	6.0 × 5.2	L	Cystic FFM	LS	NT, no VOT
29	[22]	2 d	CM in AUS at 34-GW, asymptomatic, no PM, US, a free-floating CM on the right without blood support	5.0 × 4.5	L	Absent LO and LFT, autoamputated cystic ovarian FFM in the right abdomen	LS	Hemorrhagic infarction with calcification
30	[23]	1 d	CM in AUS at 37-GW, PM in right lower quadrant, US and CT, complex CM with calcification in the right lower quadrant	6.0 × 6.0	L	Absent LO and LFT, cystic FFM attached to the omentum in the right lower quadrant	LT	Hemorrhagic infarction with calcification
31	[24]	3 d	CM in AUS after 30-GW, abdominal distention, intestinal obstruction, respiratory distress syndrome, US, complex CM	8.0	R	Autoamputated RO fixed to mesentery and terminal ileum leading to ischemia for 15 mm	LT	Hemorrhagic infarction with calcification, no VOT
32	[24]	10 m	CM in AUS after 30-GW, asymptomatic, no PM, US, complex CM	4.0	L	Autoamputated LO in retrovesical area	LT	Hemorrhagic infarction with calcification, no VOT
33	[24]	3 m	CM in AUS after 30-GW, asymptomatic, no PM, US, complex CM	5.5	L	Autoamputated LO connected with right adnexa	LT	Hemorrhagic infarction with calcification, no VOT
34	[24]	17 d	CM in AUS after 30-GW, asymptomatic, no PM, US, complex CM	2.9	L	Autoamputated LO connected to cecum with adhesions	LS	Hemorrhagic infarction with calcification, no VOT
35	[25]	4 d*	CM in AUS at 28-GW, asymptomatic, no PM, US and MR, right side CM	4.0 × 3.5	L	Absent LO, autoamputated FFM in the right side abdomen	LS	NT with small amount VOT
36	Uygun et al. (Present Case) 2012	4 y	Acute abdomen, intestinal obstruction and recurrent abdominal pain symptoms (tenderness, vomiting), US, free fluid, PXR, normal	2.8	R	Absent RO and RFT ending with band on cecum and pressuring ileum, calcified FFM	LT	NT with calcification

AUS: antenatal ultrasonography, CM: cystic mass, PE: physical examination, IVU: intravenous urography, PXR: plain X-ray, UTI: urinary tract infection, VCUG: voiding cystourethrography, FFM: free-floating mass, R: right, L: left, BL: bilateral, UL: unilateral, UK: unknown, LT: laparotomy, LS: laparoscopy, LO: left ovary, RO: right ovary, RFT: right fallopian tube, LFT: left fallopian tube, NT: necrotic tissue, VOT: viable ovarian tissue, MRI: magnetic resonance imaging, CT: computed tomography, US: ultrasonography, PM: palpable mass, GW: gestational age, d: days, w: weeks, m: months, y: years.

*Preoperative diagnosis of autoamputated ovary.
